# A Framework for Single-Panicle Litchi Flower Counting by Regression with Multitask Learning

**DOI:** 10.34133/plantphenomics.0172

**Published:** 2024-04-15

**Authors:** Jiaquan Lin, Jun Li, Zhe Ma, Can Li, Guangwen Huang, Huazhong Lu

**Affiliations:** ^1^ College of Engineering, South China Agricultural University, Guangzhou 510642, China.; ^2^ Guangdong Laboratory for Lingnan Modern Agriculture, Guangzhou 510642, China.; ^3^ State Key Laboratory of Agricultural Equipment Technology, Beijing 100083, China.; ^4^ Guangdong Academy of Agricultural Sciences, Guangzhou 510640, China.

## Abstract

The number of flowers is essential for evaluating the growth status of litchi trees and enables researchers to estimate flowering rates and conduct various phenotypic studies, particularly focusing on the information of individual panicles. However, manual counting remains the primary method for quantifying flowers, and there has been insufficient emphasis on the advancement of reliable deep learning methods for estimation and their integration into research. Furthermore, the current density map-based methods are susceptible to background interference. To tackle the challenges of accurately quantifying small and dense male litchi flowers, a framework counting the flowers in panicles is proposed. Firstly, an existing effective algorithm YOLACT++ is utilized to segment individual panicles from images. Secondly, a novel algorithm FlowerNet based on density map regression is proposed to accurately count flowers in each panicle. By employing a multitask learning approach, FlowerNet effectively captures both foreground and background information, thereby overcoming interference from non-target areas during pixel-level regression tasks. It achieves a mean absolute error of 47.71 and a root mean squared error of 61.78 on the flower dataset constructed. Additionally, a regression equation is established using a dataset of inflorescences to examine the application of the algorithm for flower counting. It captures the relationship between the predicted number of flowers by FlowerNet and the manually counted number, resulting in a determination coefficient (*R*^2^) of 0.81. The proposed algorithm shows promise for automated estimation of litchi flowering quantity and can serve as a valuable reference for litchi orchard management during flowering period.

## Introduction

Litchi is one of the most important tropical fruits in China, with high nutritional value, distinct features, stable benefits, and strong competitiveness, making its production one of the pillar industries for promoting rural poverty alleviation and wealth accumulation [1,2]. The flowering conditions and flowering period management of litchi influence its yield and fruit quality [3]. During the flowering period of litchi, the number of flowers intuitively reflects the growth status of litchi, providing information support for researchers to conduct phenotypic information studies on litchi. It is also an important indicator for a series of production management tasks, such as flowering rate determination and flowering period estimation [4–6]. Efficient and accurate information on litchi flower quantity can help enhance the informatization and intelligentization levels of orchard management and accelerate litchi phenotype research.

With the rapid advancement of computer science technology and the incorporation of smart agriculture, the utilization of computer vision in agriculture has greatly expanded [7,8]. This growth can be attributed to the decreasing costs of equipment and the development of algorithms that effectively address intricate visual challenges. In phenotypic research on flower quantity, researchers typically manually count the number of litchi flowers at the inflorescence level [9]. This method is both time-consuming and labor-intensive, but it enables the collection of time-series data throughout the flowering period. The aim is to gain insights that can contribute to the resolution of specific biological issues. Traditional flower counting typically involves manual estimation by researchers based on their experience and requires frequent observation and professional knowledge. However, this approach is time-consuming and prone to errors.

In the problem of flower counting, researchers employ diverse image sensors and algorithms to accurately count the number of flowers on various fruit trees. Typically, traditional image process technologies are applied by classifying the pixels of flower images from fruit trees with color threshold. This allows for the establishment of a correlation between the proportion or number of pixels classified as target flowers and the actual number of flowers [10–13]. However, this method is susceptible to environmental lighting and requires strict environmental control, thereby limiting its accuracy and generalizability. With the increasing popularity of convolutional neural networks (CNNs), researchers have employed numerous sophisticated models to overcome these limitations. In particular, advancements in object detection and instance segmentation algorithms have significantly improved flower identification at the image level. Consequently, researchers have collected diverse flower datasets representing various growth stages to train state-of-the-art deep CNN models with the aim of achieving precise flower recognition outcomes that ultimately determine flower counts [14–20]. Obtaining count results based on detection methods requires complex network model structures, involving regional proposal estimation and non-maximum suppression, thus increasing computational complexity and memory requirements. Additionally, annotating training samples in images containing multiple flowers using bounding boxes or polygon annotations prolongs the manual annotation time.

To address these concerns, researchers have proposed regression-based models for flower counting, which involve inputting RGB images into a network model to directly estimate the number of flowers. Lu et al. [21] introduced TasselNet, which demonstrates strong adaptability to field changes by simulating local visual features of field images and regressing on local counts of corn ears. The model is based on the long and thin characteristics of maize tassels, and compared with the collection method of litchi flower, its collection method of overlooking image has less background interference. Bhattarai and Karkee [22] developed CountNet, a weakly supervised flower/fruit counting network based on deep learning. Unlike previous approaches, CountNet learns from image-level annotations and directly obtains the number of objects by using target object-containing RGB images as input, which simplifies the detection process. However, this method ignores the spatial distribution of targets, making it impossible to distinguish the source of their quantity results.

Moreover, researchers have utilized multispectral cameras to capture images of fruit trees during their flowering periods. By employing supervised classification methods to separate flower pixels in the images, they have conducted correlation analyses between the estimated distribution of flowers and final yield distributions. Researchers have also used hyperspectral imagers to investigate prediction results for flower quantity per unit area by extracting characteristic wavelengths [23–25]. This kind of method is more direct, but the sensor measurements are susceptible to environmental factors, particularly lighting, and the sensors are relatively costly.

The preceding review presented an overview of various flower counting methods, but research specifically focused on counting litchi flowers remains limited. Xiong et al. [26] utilized a deep semantic segmentation network to categorize the pixel regions of litchi fruit trees into flowers and leaves, aiming to predict flowering intensity. However, this method only allows for a qualitative prediction of flowering intensity and is susceptible to spatial influences during image capturing. Lyu et al. [27] utilized the method of multi-teacher pre-activation feature distillation for the detection of litchi flowers on an embedded platform. This approach is able to accurately accomplish the detection of litchi flowers. However, it is achieved by removing samples of litchi flowers from trees, scattering them on a structured background, and taking photographs, which is inconsistent with the goal of directly estimating the non-structured litchi flower on the tree. Ye et al. [28] also employed object detection techniques to address the challenge of detecting closely clustered and occluded litchi flowers. They modified the bounding box and predicted box positions using a polyphyletic loss function to minimize missed detections. However, this approach still struggled to effectively detect litchi flowers in densely overlapping scenes. To address the problem of difficult counting since litchi trees have small flowers that bloom in large numbers, Lin et al. [29] proposed a litchi flower counting method that utilizes density map regression. They investigated techniques for constructing flower count density map datasets and trained multiple column CNNs to directly obtain litchi flower count density maps from RGB images. However, this method relies on pixel-level regression statistics, which may render it vulnerable to interference from the background during counting.

The density map-based approach demonstrates advantages in estimating both small and massive litchi flower. When applying density map-based methods to the task of counting litchi flowers per panicle, 3 specific problems need to be awarded. First, in the phenotype research of litchi flower, a single panicle is often taken as the research object, which allows researchers to understand the growth state of trees. Second, the results obtained from density map-based methods can exhibit significant variation when images are captured at different distances. This variation poses a challenge to achieving consistent and accurate flower counts. Third, due to the pixel-level regression characteristic of the method, the presence of background in the image can easily disrupt the counting results.

To overcome these challenges, a framework for flower count aiming to quantify single-panicle flower quantity more accurately is proposed. To locate the single panicle, existing advanced instance segmentation network was introduced for the coarse detection of litchi panicles, which segmented the panicles in images. Then, a novel multi-task learning algorithm FlowerNet based on semantic information and density maps was developed to accomplish flower counting task. In the algorithm, a VGG16 backbone with panoptic feature pyramid networks was adapted to address the challenge of scale variation, in which the feature maps of different scales were generated to obtain the strong and weak semantic information in the image with the addition of the convolutional and pooling layers. The panoptic feature pyramid module was introduced to process different scale information. To address the challenges of complex background interference, the strategy of multi-task learning was applied. A density mask generation network was designed to characterize image semantics and the density map counting task. It well excluded non-target regions and completed the density map generation task though the optimization of the losses for these 2 tasks. In the final prediction process, the jointly generated binary mask of flower panicles and the predicted density map were used to predict the final flower quantity density map and obtained the predicted total flower quantity of the image. Finally, the noise of background was successfully suppressed, and a high-quality density map was generated.

Furthermore, to improve the accuracy of the prediction of the flower number during the early flowering stage, the relationships of flower numbers between observed, labeled, and predicted by the FlowerNet were investigated for the individual flower panicles. A linear fitting regression equation was established, relating the flower count predicted by FlowerNet to the actual flower count obtained by manual counting. This reduced the error in the final predicted flower count from a single perspective and validated the obtained phenotypic data by comparing them with manually counted data.

The major contributions of this paper are summarized as follows:

1. An effective convolutional framework for quantifying the litchi flower in a panicle is proposed on RGB images at a single perspective, which leverages the segmentation of panicles and the counting in panicles.
2. A multiscale module has been designed to facilitate the extraction and fusion of features at various scales, enabling adaptation to differences in information across scales.
3. A multi-task learning strategy is introduced to perceive the semantical information of images and density map generation, and semantic division and counting tasks are finally completed, which restrains the intervention of background.
4. The relationship between the actual flower count and the predicted flower count by FlowerNet is explored, which effectively improves the accuracy of flower number in panicles at a single angle.

## Materials and Methods

### Statistical analysis of datasets

#### Dataset for model evaluation

**Fig. 1.**
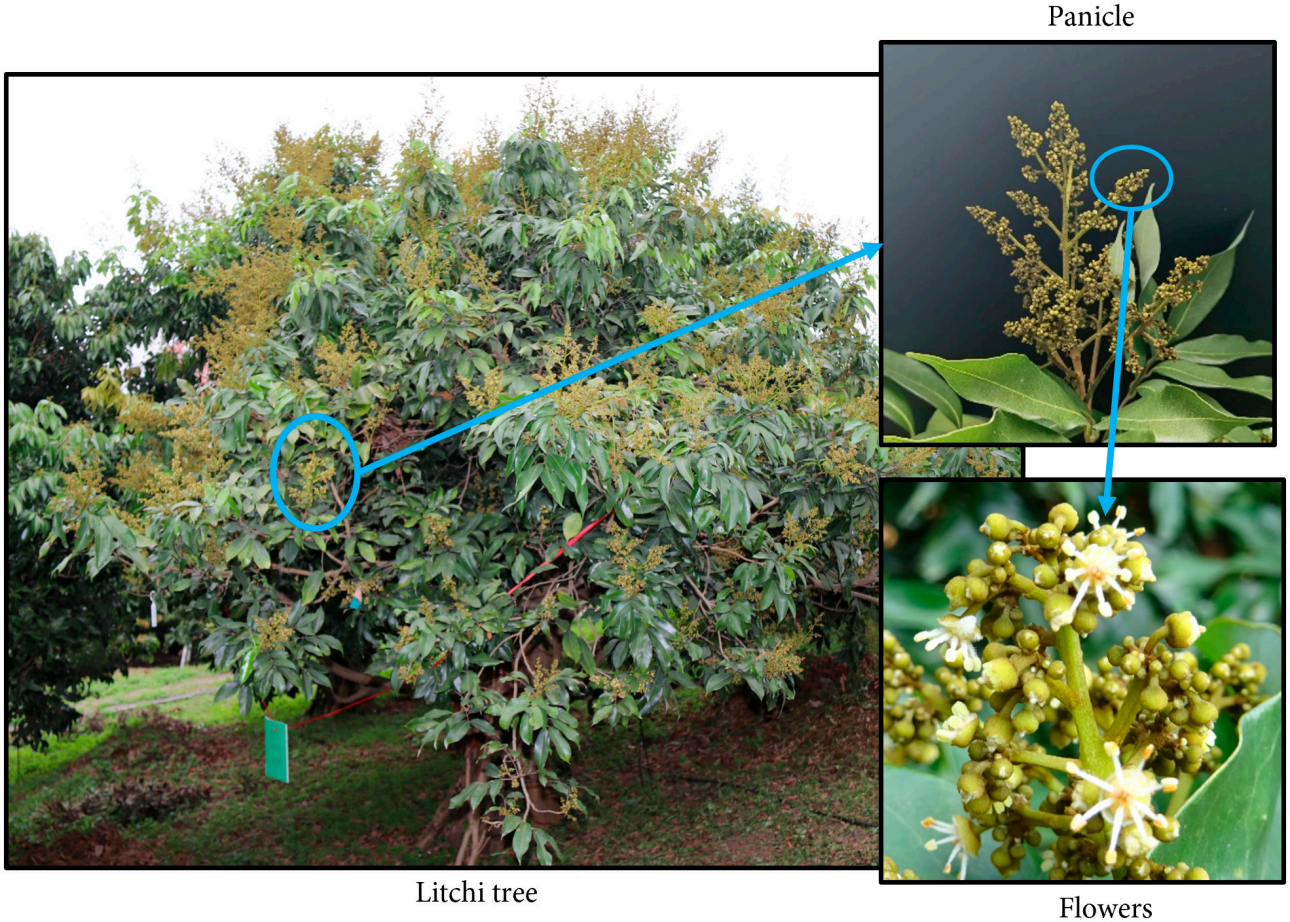
The overview of the litchi tree and its flower morphology.

The experimental data used in this study were obtained from a litchi resource orchard at the Institute of Fruit Tree Research, Guangdong Academy of Agricultural Sciences. The orchard has an area of 0.12 hm^2^. To collect images, 1 to 2 representative sampling points were randomly selected. In this experiment, 2 litchi flower varieties, Guiwei and Feizi Xiao, were selected as research objects to enrich the dataset to facilitate the creation of benchmarks, whose flower morphology has no great difference. To ensure the diversity of the data samples, images of the first-period male litchi flowers during the early flowering stage were taken from different angles and distances at different times under different environmental lighting conditions, including from 9:00 to 10:00 AM and from 5:00 to 6:00 PM. At this stage, the litchi tress starts flowering, and then it forms a beautiful cluster of flowers, called a panicle, at the end of the branches. The panicles are conical in shape and have small yellow-green flowers. A fully developed panicle consists of a main stem and several lateral branchlets. The flowers on the panicles have different stages of blooming. Initially, they are in the bud stage. After complete development, the stamens of the male flowers are slender, varying in length, usually with 7 to 9 filaments connected to the base, and the anthers with pollen are attached to these long filaments, as shown in Fig. 1. The collected data were image data generated from flower phenotypic traits during the full flowering period of litchi, which included different morphologies of male litchi flowers, making up dataset for instance segmentation and flower counting.

**Fig. 2.**
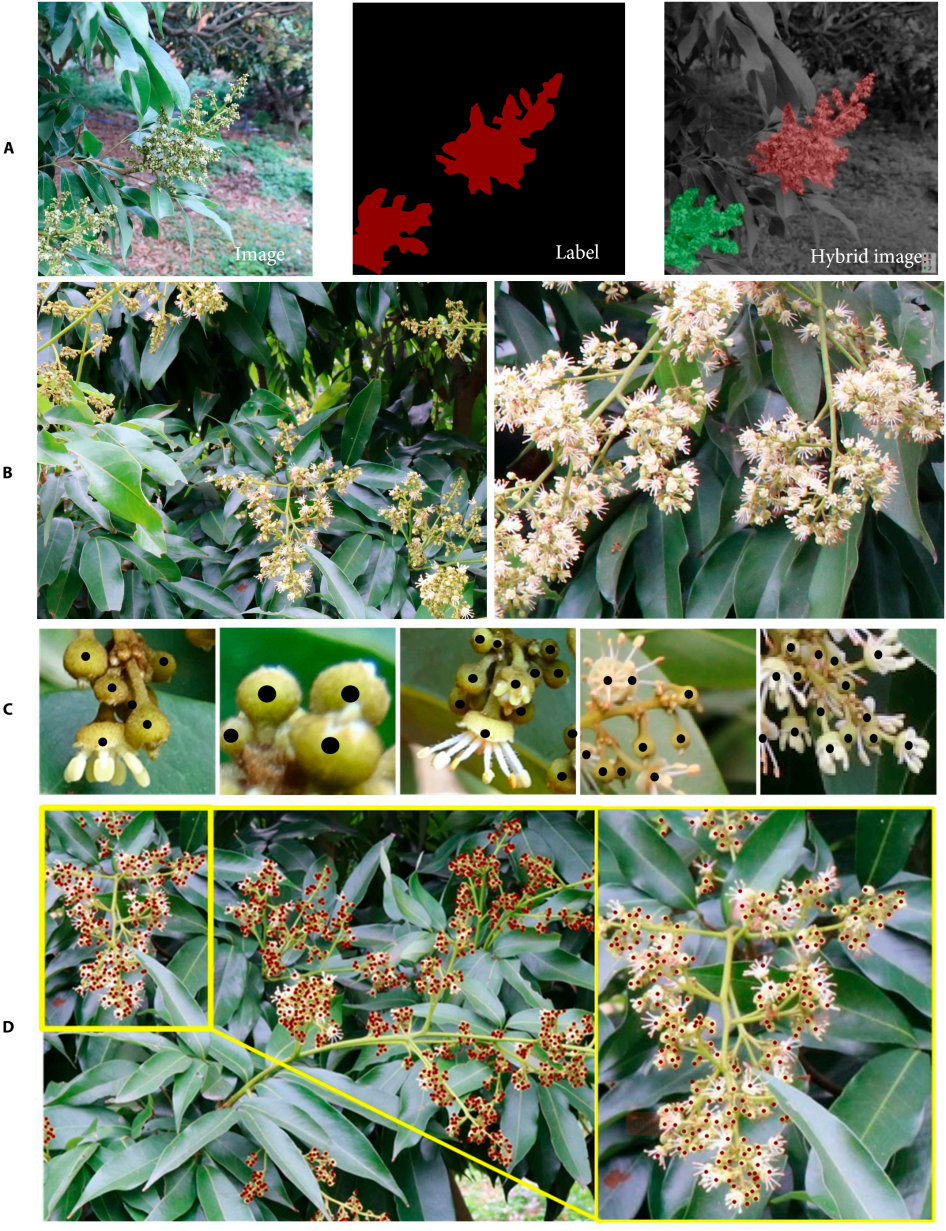
Example of images and the annotations of instance segmentation and density map. (A) Example of the annotation of panicles. (B) Litchi flower images. (C) Example of different typical annotation situations. (D) Example of complete annotation results.

To address the issue of flower counting, with a focus on the main subject being single panicle, we introduced an instance segmentation model to differentiate panicles during the initial flowering stage. To prepare the dataset for the instance segmentation algorithm, it is essential to obtain ground truth masks for the litchi flower panicles. In the actual situation, the presence of intertwined branches and unclear edges within the flower panicles, such as hollow regions and blurred boundaries, may lead to less precise segmentation results. However, the primary purpose of extracting the flower clusters is to estimate the individual flower quantities, and the segmentation results do not need to be highly accurate. Therefore, some simple specifications for labeling are made. Through the annotation software, the region of each panicle is simply divided in the original image, and the masks are obtained. The hybrid image of the original image and the mask is shown in Fig. 2A, where different colors represent different instances of panicles. As shown in Fig. 2A, for cases where the edges of the flower panicles are not clear during the annotation process, it is acceptable to appropriately expand the scope. For cases where the flower panicle branches intersect, the mask can include non-flower cluster areas with hollows inside them.

Regarding the preparation of the flower quantity counting dataset, numerous 1,024×768 resolution images were randomly cropped from the original images with a resolution of 6,000×4,000. The collected image samples are shown in Fig. 2B. Eventually, during the image annotation stage, the Litchi male flowers were point-annotated using LabelMe software [30], following Lin’s annotation method [29]. The flowers in the images exhibited diverse forms resulting from their natural growth patterns. Therefore, the annotation process was consistently conducted at the convergence point of the filaments, which was positioned at the center. After the completion of annotation, generated Json-format files stored the image location information of the target objects in the images. The typical annotation situation is shown in Fig. 2C, which includes the cases of all the flowering stages and occlusion. The complete labeling situation is shown in Fig. 2D.

Finally, both datasets required for the framework model have been completed, and the specific details are shown in Table 1. Num represents the number of images in the dataset. Train represents the number of images in the training set. Test represents the number of images in the test set. Total represents the total number of flowers in the dataset. Max represents the maximum flower number in an image. Min represents the minimum flower number in an image. Avg represents the average flower number per image.

**Table 1.** Detailed information on the flower datasets

Dataset	Num	Train	Test	Total	Max	Min	Avg
Counting	337	268	69	173,949	1,504	40	516.16
Segmentation	242	194	48	–	–	–	–

#### Single-panicle counting dataset

**Fig. 3.**
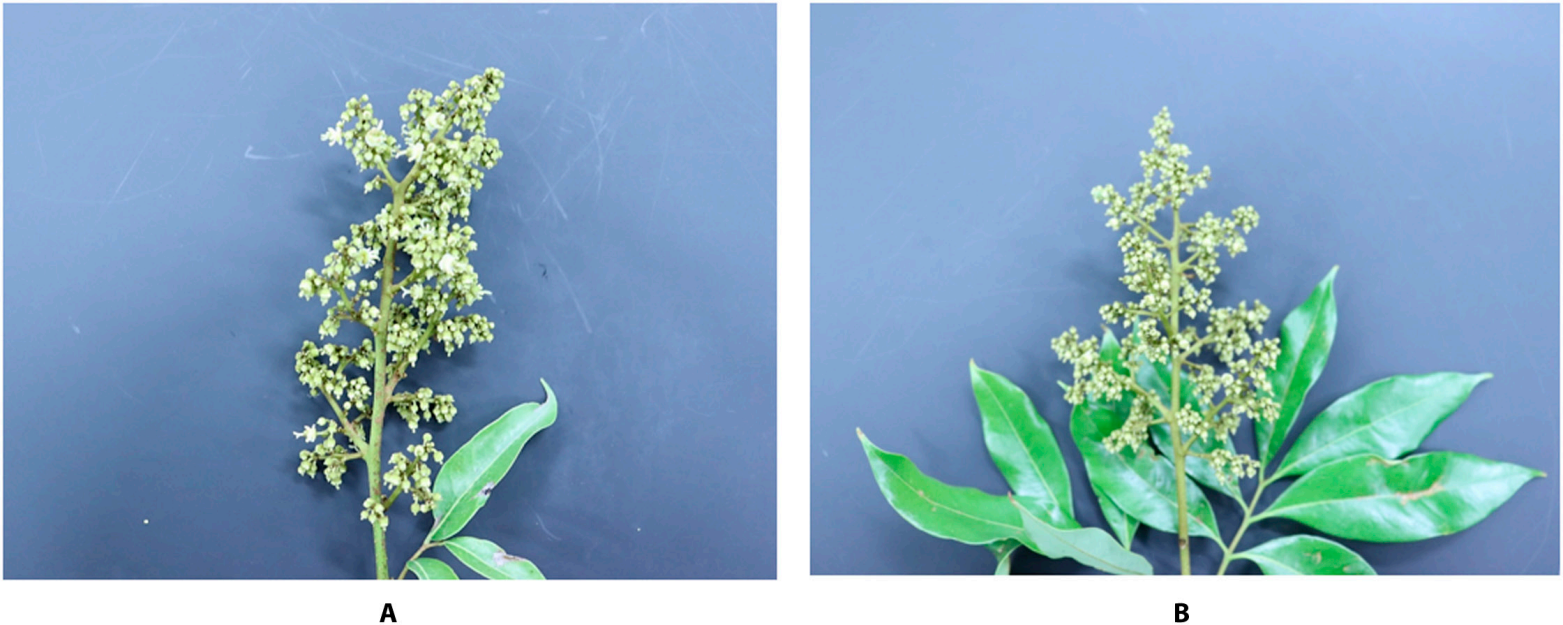
Examples of single panicles. (A) and (B) are images captured for network predictions.

To investigate the practical applications of flower quantity prediction from images and enhance its accuracy, this section focuses on single panicles and collect panicle image data. A fixed color plate (50×50 cm in area and 2 mm thick) was placed on one side of a panicle as a background for the collection of FeiziXiao flower panicle images. The collection time was from 3:00 to 5:00 PM during the initial flowering period of FeiziXiao in sunny weather, and the image resolution was 1,024×1,024. To obtain a complete image of the panicle, the panicle was placed in the middle area of the fixed background board. The collected image included the entire background board and one side of the panicle, as shown in Fig. 3. The collection results are shown in Table 2, and the corresponding image flower quantity information was annotated. In addition, 58 images were collected to assess the correlation between the number of flowers in a single flower panicle predicted by the FlowerNet algorithm after training and the actual number of flowers obtained by manual counting. 

**Table 2.** Information on the single-panicle counting dataset. Type represents different ways to collect flower number, where Observation means the flower counting by hand and Annotation means the flower annotated result. Num represents the number of images in our single-panicle counting dataset. Train represents the number of images split to train set. Test means the number of images split to test set. Total means the number of flowers in the whole dataset. Max means the maximum flower number in an image. Min means the minimum flower number in an image. Avg means the average flower number in an image.

Type	Num	Train	Test	Total	Max	Min	Avg
Observation	110	99	11	52,235	1,022	144	474.86
Annotation				32,106	687	71	291.87

### Single-panicle flower counting framework

**Fig. 4.**
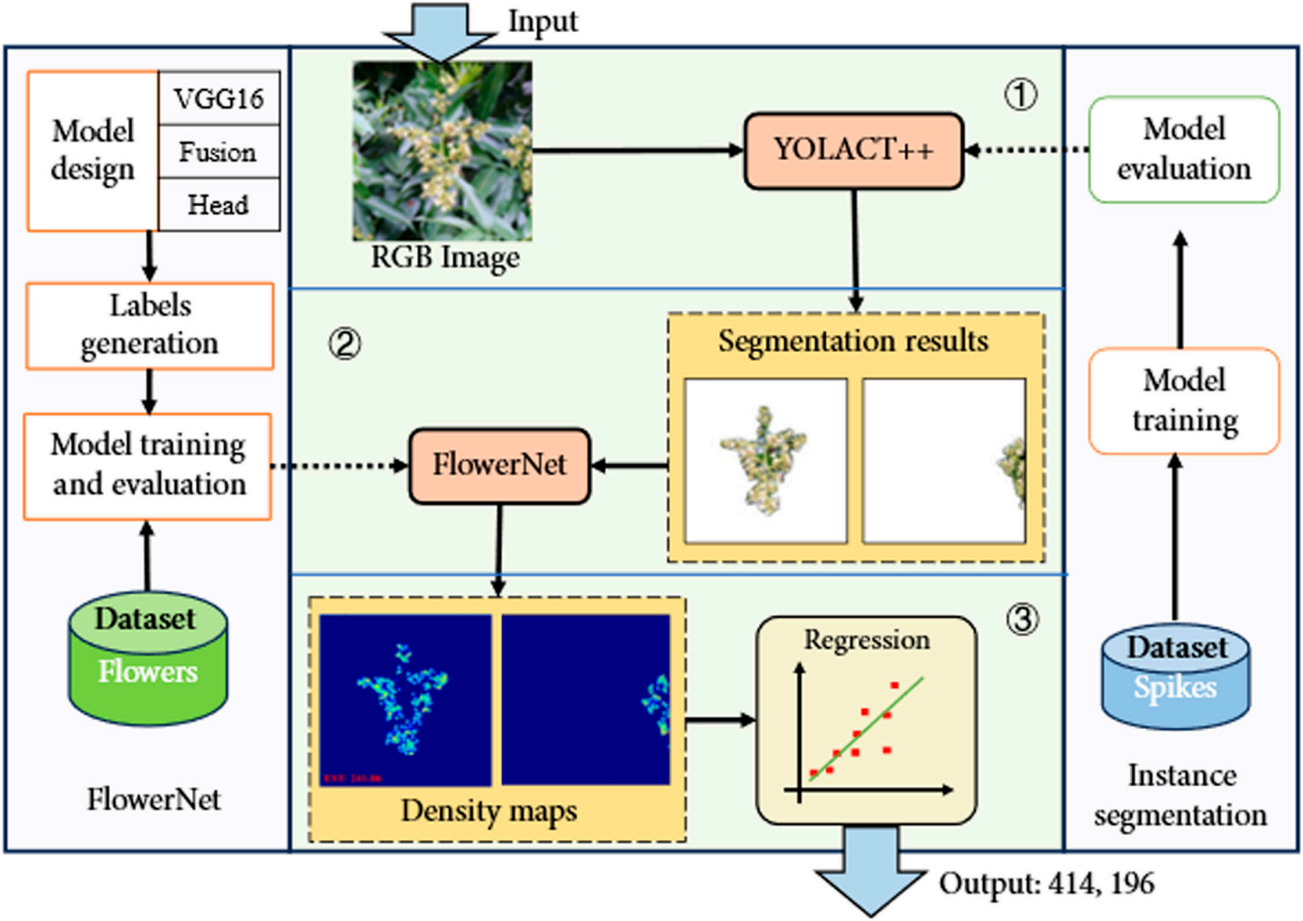
Overall framework for single-panicle flower counting.

This study presents a framework for quantifying the number of litchi flowers in a panicle based on analysis of RGB images. The framework comprises 3 main components, as shown in Fig. 4. The initial part involves the extraction of individual flower panicles, intending to obtain the flower panicle mask from the RGB image and extracting images in the corresponding regions. To accomplish this, advanced instance segmentation models are employed, specifically the YOLACT++ [31], which is renowned for its stability and efficiency in the extraction process. However, due to the inherent uncertainty associated with the edges of the flower panicles, achieving precise edge masking poses a challenge. As a result, this process only yields a rough extraction of the individual flower panicles. The second part introduces a novel algorithm for counting the quantity of flowers based on density maps. This algorithm specifically focuses on counting segmented flower panicles. It leverages deep learning feature extraction to share image feature information and utilizes different convolution branches to optimize distinct loss functions, enabling targeted learning. Ultimately, this algorithm integrates the semantic information of the flower panicles to achieve both semantic differentiation and accurate flower quantity counting. The last part of this framework involves using a regression method to map the flower quantity obtained by the algorithms on RGB images to the actual flower quantity. Experimental studies are being conducted to obtain the real flower quantity and reduce the error in flower quantification.

### The flower counting network FlowerNet

In this section, the structures of various parts of the proposed baseline network are first introduced, including the feature extraction network, the multiscale feature fusion network, and the head network of the density mask generation network (DMN). The model is then optimized using a combined loss function. Finally, the structure of the FlowerNet model is fully described.

#### Backbone network

VGG16 is a 16-layer deep CNN developed by the Visual Geometry Group Laboratory of the University of Oxford. It achieved outstanding results in the 2014 ILSVRC image classification competition. VGG16 successfully constructs a deep CNN by repeatedly stacking 3×3 convolutional kernels and 2×2 max pooling layers. This architecture is simple, easy to implement, and can achieve high performance through pretraining on large image datasets. Using VGG16 as the backbone feature extraction network enables the extraction of both low-level and high-level features in images, which is crucial for computer vision tasks. Moreover, its simplicity and performance make it adaptable for integration into networks of various types. VGG16 also demonstrates excellent transfer learning capabilities, enabling the use of limited computing resources and saving considerable time. In addition to its strong feature extraction capabilities, VGG16 exhibits rapid convergence, effectively addressing the issue of overfitting with limited training images. As a result, it is widely favored for numerous counting tasks.

**Fig. 5.**
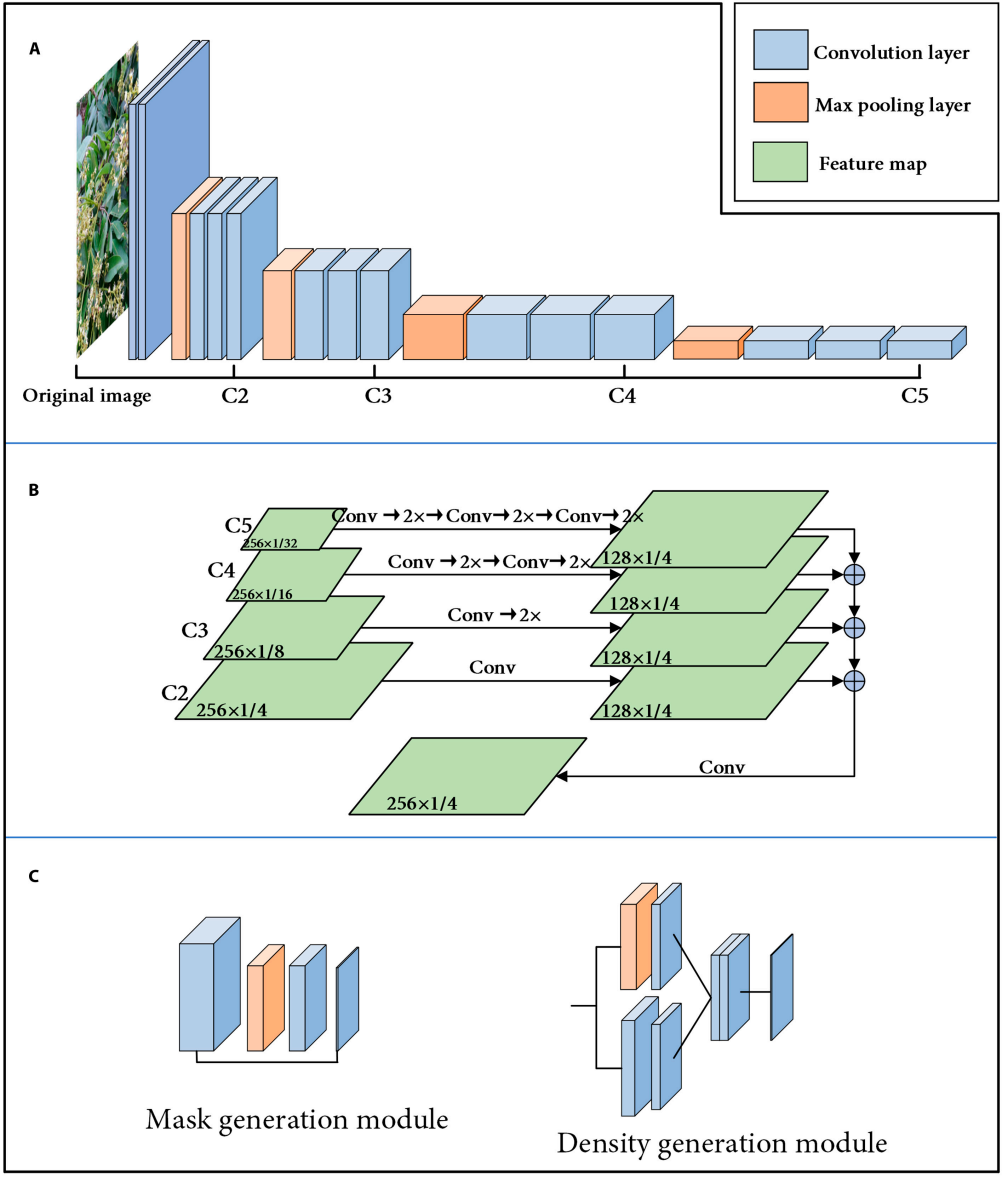
The structure of the parts of the network. (A) The structure of the backbone network VGG16. (B) The structure of panoptic feature pyramid networks. (C) The detailed structure of the DMN.

In this section, the first 13 layers of VGG16 are chosen as the backbone feature extraction network, and the fully connected layers used for image classification tasks are removed, as shown in Fig. 5A. The purpose of this is to investigate the impact of multiscale feature maps produced by various convolutional blocks on the subsequent feature fusion network. This study includes these feature maps (C2, C3, C4, and C5, as shown in Fig. 5A) in the fusion network for exploration. C2, C3, C4, and C5 represent different output feature maps at different network scales, with each output feature map being generated before the network performs down-sampling operations. Specifically, C2 denotes a feature map comprising 128 channels following 2 convolution operations, C3 denotes a feature map comprising 256 channels following 3 convolution operations, C4 denotes a feature map comprising 512 channels following 3 convolution operations, and C5 denotes a feature map comprising 512 channels following 3 convolution operations. The backbone network utilizes convolution operations with the same kernel size of 3×3.

#### Multiscale feature fusion

To fuse information from shallow and deep features of different scales and to solve problems posed by the imbalance of semantic information and the lack of effective information sharing mechanisms in different network layers, a feature fusion module is added after the output of different-scale feature maps in the main feature extraction network. This is done to use features of different scales and levels to obtain richer and more effective feature expressions, thereby improving the model’s performance and robustness. Inspired by the paper “Panoptic feature pyramid networks” [32], the omnidirectional feature pyramid model is used to share feature information output by VGG16 at different scales, making the fused multiscale information more detailed. By utilizing the advantages of the feature pyramid network, high-quality features can be extracted at multiple scales, and most of the computational resources are shared.

The panoramic feature pyramid model first uses a 1×1 convolutional layer to unify the feature maps (C2, C3, C4, and C5) of different channels and different scales, which are output by the backbone feature extraction network into the same 256-channel feature maps, *Y*_2_, *Y*_3_, *Y*_4_, and *Y*_5_. Features are extracted through parallel branches operating on features of multiple scales. This approach maintains consistent sizes and channels of the feature maps across all scales, promoting integration at various levels of resolution. An addition operation is performed on the unified feature maps to achieve the complementation and enhancement of different features, and this process can be expressed as follows:

$$Y=f_{1\times 1}\left(f\left(Y_{2}\right)+\sum_{i=3}^{5}Y_{i}^{n}\right)$$ (1)

Yin=Yi,n=2FYin−1,n>2 (2)

$$F\left(x\right)=\mathrm{up}\left(f\left(x\right)\right)$$ (3)

where the function *up* represents nearest-neighbor doubling sampling, the function *f* represents the 3×3 convolutional layer imposed on the multiscale feature map, *f*_1 × 1_ represents the convolution operation of the kernel of size 1×1 applied after the addition and fusion of the network, *Y* represents the result after model addition, and *Y_i_* represents the feature map of the *i*th layer. The specific network structure is shown in Fig. 5B.

Unlike the original model, which uses the number of output channels from the panoramic segmentation category as the feature output, the number of channels output by this network model through 1×1 convolution is set to 256. This value is used for the fusion of feature information after switching the multiscale feature scale to channels. A detailed analysis of the significance of this channel value will be conducted in subsequent ablation experiments.

#### Head network

In the practical application of litchi inflorescence counting, various factors, including illumination, background leaves, and noise, often affect the accuracy of flower counting. This becomes particularly challenging against complex backgrounds, where the model’s counting becomes more difficult and its robustness is reduced, leading to increased counting errors. Importantly, even in the density map of the predicted results, there may be instances of counting values in the background areas. These counting results can interfere with the overall count of litchi flowers after integration, thereby impacting the network’s judgment.

Multitask learning is a technique in machine learning that leverages shared network layers and parameters across tasks to train a neural network model to concurrently perform multiple tasks. This approach improves the model’s generalization performance by utilizing shared information among tasks, enabling the acquisition of more effective and concise features. Consequently, to overcome technical errors resulting from background factors, the current section adopts a multitask learning strategy to develop a density mask generation network specifically designed to isolate flowers from their background in images, thereby enabling more efficient and accurate counting. The DMN is positioned at the head output network of the overall architecture, as depicted in Fig. 5C, and comprises 2 modules: a mask generation module and a density generation module. The former is responsible for generating the mask of flowers and background, while the latter focuses on producing the density map for flowers.

During the training process, the mask generation module is utilized to acquire semantic information from the image and differentiate between foreground and background features. This module generates a binarized mask specifically highlighting the inflorescences. The density generation module is responsible for solving the ultimate density estimation task. To enhance the receptive field and feature map diversity, this module incorporates an attention mechanism derived from YOLOv4 [33], called the max-pooling (MP) module. By implementing nonaligned pooling operations, the MP module improves the feature map. Consequently, it generates a flower density map for accurate counting. Both modules contribute to the tasks of identifying foreground–background differences and performing density counting.

**Fig. 6.**
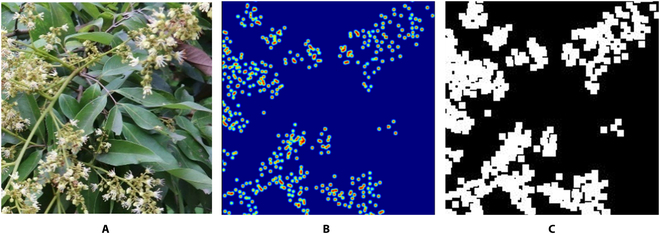
Examples of 2 labels for the network to learn. After the RGB image (A) input, the DMN network learns to reduce the difference between the predicted result and the output density map (B) and the corresponding mask (C).

Figure 6 presents these 2 labels for the network to learn. Figure 6B shows the density map that the density generation module learns to obtain while training, which helps the network to purposefully perform the task of counting the number of flowers. Figure 6C displays the target mask while training the mask generation module, which helps the network to specify the categories of each pixel in the image and then learn the semantic context. During the prediction phase, the binary mask and density map are combined through elementwise multiplication. The final density map outcome is determined through matrix multiplication. Assume that *x_mask_*(*i*, *j*) refers to the element in the *i*th row and *j*th column of the binary mask and that *x_mask_*(*i*, *j*)∈0, 1. An *x_mask_*(*i*, *j*) value of 1 indicates that the class predicted for the corresponding pixel point is the flower class, and an *x_mask_*(*i*, *j*) value of 0 indicates that the class predicted for the corresponding pixel point is the background class. Therefore, the final flower density map can be represented by the following formula:

$${\hat{y}}_{\text{density}}\left(i,j\right)=\left\{\begin{array}{cc} y_{\text{density}}\left(i,j\right) & x_{\text{mask}}\left(i,j\right)=1\\ 0 & x_{\text{mask}}\left(i,j\right)=0 \end{array}\right.$$ (4)

In the formula, *y_density_*(*i*, *j*) is the value in the *i*th row and *j*th column of the density map and$\;{\hat{y}}_{\text{density}}\left(i,j\right)$ is the value in the *i*th row and *j*th column of the final flower density map.

#### Combined loss function

This section uses a multitask learning approach to optimize the model for flower counting, with the aim of addressing the task of distinguishing foreground and background through the acquisition of semantic information, as well as tackling the challenge of obtaining flower density maps by learning higher-level features. To effectively address both tasks using shared model parameters, a custom loss function is needed. The combined loss function consists primarily of the mask loss and density map regression loss. The mask loss is computed as the cross-entropy between the predicted foreground–background mask and the actual label, making it suitable as a loss function for binary classification. The density map regression loss, along with other counting loss functions that rely on density map regression, employs the Euclidean distance to measure the disparity between the estimated density map label and the actual density map. By incorporating these 2 loss functions into the final output layer and utilizing the derivative chain rule, the partial derivatives of each loss with respect to each parameter layer can be calculated. The resulting values are then multiplied by a learning rate to obtain the parameter update quantity, which facilitates weight adjustments and the acquisition of relevant parameters by the model. The definitions of these 2 loss functions are provided below:

$$L_{\text{mask}}=\frac{1}{N}\sum_{i=1}^{N}L\left(P_{i},{\mathrm{GT}}_{i}\right)$$ (5)

$$L\left(P_{i},GT_{i}\right)=-\sum_{m=1}^{H}\sum_{n=1}^{W}\left[gt_{\mathrm{mn}}\times p_{\mathrm{mn}}+\left(1-gt_{\mathrm{mn}}\right)\left(1-p_{\mathrm{mn}}\right)\right]$$ (6)

$$L_{\text{regression}}=\frac{1}{N}\sum_{i=1}^{N}{\left|F\left(x_{i};\theta \right)-F_{i}\right|}_{2}^{2}$$ (7)

In the formulas, *L_mask_* represents mask loss, *N* is the number of training samples, and *L*(*P_i_*, *GT_i_*) represents the loss value of the predicted mask and the label for the *i*th image. *P_i_* refers to the binarized mask predicted for the *i*th image, *GT_i_* represents the corresponding binarized ground truth mask for the *i*th image, *W* is the width of the input image, *H* is the height of the input image, *gt_mn_* is the pixel value of the input image in the *m*th row and *n*th column, and *gt_mn_* ∈ {0, 1}, *p_mn_* is the predicted pixel value of the predicted image in the *m*th row and *n*th column. *L_regression_* represents the density map regression loss, *F*(*x_i_*; *θ*) is the predicted density map of the *i*th image, and *F_i_* represents the corresponding density map label. Finally, combining the mask loss and density map regression loss yields the final combined loss function for jointly optimizing the network model. It is represented as

$$L=L_{\text{mask}}+\alpha \times L_{\text{regression}}$$ (8)

In the training process, by setting *α*=8, the balance between the mask loss and density map regression loss on the counting model is maintained to enhance robustness. Subsequent ablation experiments will further explore the optimal value of *α*. To avoid repetition, this will be discussed in detail later.

#### FlowerNet architecture

**Fig. 7.**
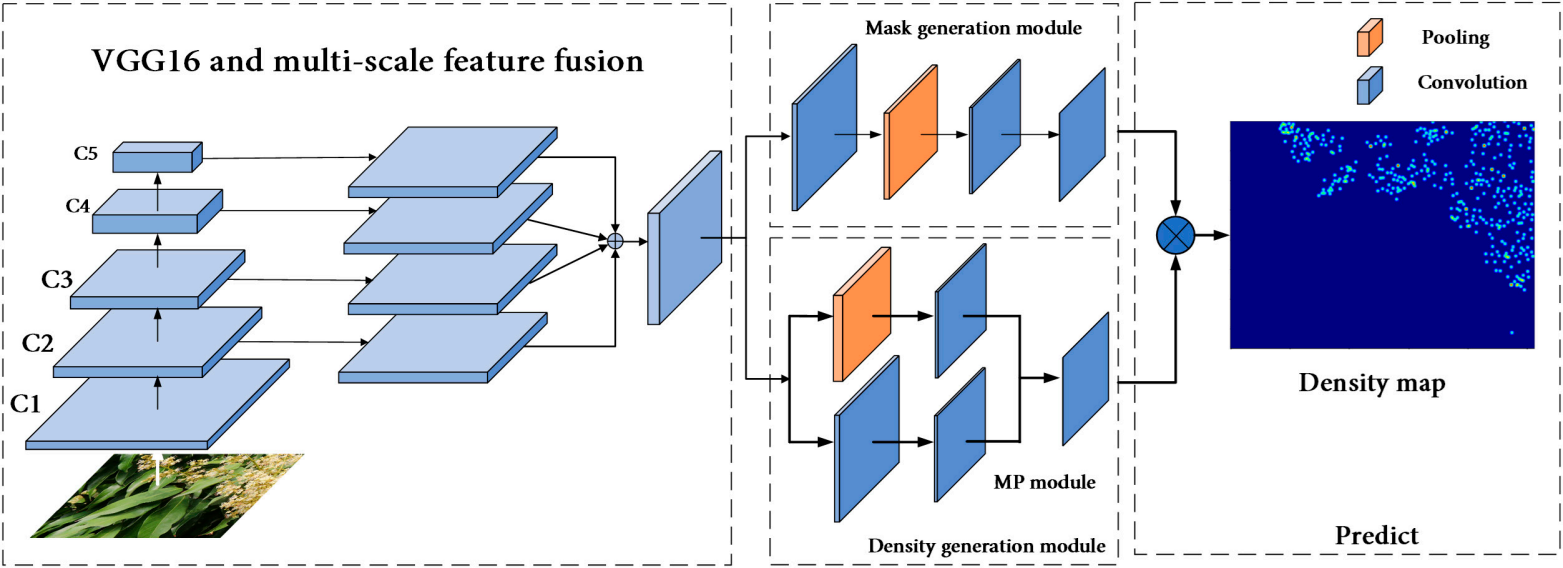
The architecture of FlowerNet.

The overall architecture of the network is shown in Fig. 7, and it is named FlowerNet. This network first passes the input image into the main feature extraction network VGG16 to extract features of different scales. Second, it uses a panoramic feature pyramid to add feature maps to fuse features of different scales in the middle, blending learned low-frequency information with high-level semantic details, realizing the sharing of shallow and deep feature map information. Finally, the fused feature map is passed to the density mask generation network. The mask generation network uses convolution for down-sampling, completing the task of separating foreground and background differences. The density generation network uses the MP module for down-sampling to complete the density counting task. In the final prediction process, the generated panicle binary mask and the predicted density map are combined to predict the final floral density map, thereby obtaining the overall floral quantity prediction result for the image.

### Label generation

The network model relies on multitask learning, which requires the generation of task labels during the learning process. This enables the loss function to optimize the comparison between the predicted and corresponding label results, thereby minimizing the discrepancy with the label values. In the present task, the network is utilized to predict the mask and density map. Consequently, it is essential to generate binary ground truth masks for the background and density map labels.

For generating density map labels for the number of flowers, a 2-dimensional Gaussian distribution with a fixed variance is used to represent the annotated target flowers [34]. This fixed Gaussian kernel simply blurs the label points of each flower. For example, let the location of each flower be *x_i_*, its distribution in the image is represented by a Gaussian kernel *G_σ_*(*x_i_*), and its corresponding location is represented by *δ*(*x* − *x_i_*). Then, the image corresponding to *N* flowers can be represented by Eq. 9. To transform this function into a continuous density function, it is convolved with the Gaussian kernel. The resulting density map can be expressed using Eq. 10. The fixed standard deviation *σ* used in this experiment is 4.0. By storing the information of the flower positions marked in each image in the Json file, a dot matrix is generated, and the density map is obtained after the above 2-dimensional Gaussian blurring.

$$H\left(x\right)=\sum_{i=1}^{N}\delta \left(x-x_{i}\right)$$ (9)

$$F\left(x\right)=H\left(x\right)*G_{\sigma }\left(x_{i}\right)$$ (10)

After generating the density map labels, they undergo thresholding. Pixels with labels greater than 0 are assigned a value of 1, indicating the litchi flower foreground in the binary mask. Pixels with labels equal to 0 in the density map are assigned a value of 0, representing the background of the mask. This process yields a binary ground truth mask created from the density map. The procedure is outlined in detail as follows:

$${\hat{\mathrm{gt}}}_{\mathrm{ij}}=\left\{\begin{array}{cc} 0 & {\mathrm{gt}}_{\mathrm{ij}}=0\\ 1 & {\mathrm{gt}}_{\mathrm{ij}}>0 \end{array}\right.$$ (11)

In the above, *gt_ij_* represents the pixel value in the *i*th row and *j*th column of the label density map, with *i*≤*H* and *j*≤*W*, where *H* is the height of the density map and *W* is its width. In addition, 1 represents the foreground of the binary mask, and 0 represents the corresponding background.

By using the density map to generate a mask, the ground truth masks can be efficiently obtained. However, because the shapes and sizes of litchi inflorescences vary, a density map filtered by a Gaussian kernel with a fixed variance cannot properly encompass the entire flower. When converting to a ground truth mask, it is easy to end up with a mask smaller than the actual flower. To generate ground truth masks more accurately, a finer method is adopted to perform morphological dilation on the converted binary ground truth masks. The edges of the ground truth masks are enlarged. The specific operation is to use a fixed-sized structuring element to scan each pixel of the mask image. On pixels overlapping with the binary image and the structuring element, an AND operation is performed. If all pixels have a value of 0, the result for the pixels is 0; otherwise, the result is 1, which results in a binary mask expanded by one round. The specific process is as follows:

$$M⊕u=\left[z|\left[\left({\hat{u}}_{z}\right)\cap M\right]\subseteq M\right]$$ (12)

**Fig. 8.**
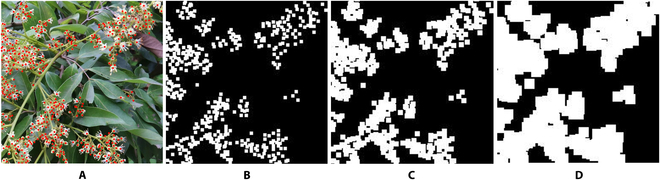
Example of ground truth masks with different structuring element sizes. (A) is the annotated result of the original image. (B) is the mask result without fine-tuning, while (C) and (D) are the masks of the expansion result using structure elements of size 3×3 and 5×5, respectively.

In the above, *M* is the ground truth mask used for dilation, *u* is a fixed-size structuring element, and *z* represents the position in the scanning process. This operation indicates that the dilation of *u* with respect to *H* is the collection of all displacements *z* in the intersection of the structuring element *u* and *H*. The width of this dilation operation is controlled by the structuring element used. The size of the structuring element affects the volume of the foreground dilation. Therefore, the quality of the generated ground truth mask can be explored by adjusting the size of the structuring element. In the subsequent ablation experiment, the optimal size of the structuring element will be further explored. Figure 8 shows examples of ground truth masks with different structuring element sizes.

### Flower quantity estimation

When utilizing FlowerNet to estimate the visible flower count of an individual panicle, a univariate linear model is employed to refine the final flower count of the panicle. This is expressed mathematically as *y* = *ax* + *b*, where *y* denotes the number of flowers obtained by manually counting the flowers in a single panicle and *x* represents the number of flowers predicted by FlowerNet. The utilization of a linear regression model presents a straightforward approach to estimating the actual number of flowers of paniclelets depicted in images. Specifically, the number of flowers of a panicle predicted by the FlowerNet algorithm serves as the input for the linear model.

### Experimental design

This paper uses a training validation strategy based on image patches and a testing strategy based on complete images. To enhance the network’s ability to adapt to flower images of varying scales and densities, as well as expedite the convergence of the model, each sample in the training phase is divided into 9 distinct image blocks. These blocks serve as the grayscale image inputs for the network. In the testing phase, the model is tested using complete images of different resolutions, which are directly input into the model. The prediction operations for both our model and the comparison models in this study are performed on a graphics processing unit (GPU) server. The experimental configuration is detailed in Table 3. To mitigate the issue of gradient vanishing or explosion during the forward propagation of the model, the model weights are initialized uniformly. The Adam optimizer is employed for gradient descent. The efficacy of the proposed module on the dataset is assessed, and several rounds of ablation experiments are conducted.

**Table 3.** Experimental environment configuration

Configuration	Parameter
CPU	Intel Core i9-10980XE
GPU	Nvidia RTX A6000
System	Ubuntu 18.04
CUDA	11.4
PyTorch	1.13

There is currently no universal benchmark dataset for counting litchi flowers, which hinders the development and evaluation of flower counting models. In order to establish a baseline for our experiments, we assembled a collection of flower counting datasets. The MCNN has gained popularity as a benchmark model in crowd counting due to its high performance and low computational cost and has been widely adopted for various counting problems. Specifically, Lin et al. employed MCNN as a reference model for comparing the performance of litchi flower counting. In evaluating our FlowerNet model, we have chosen MCNN as the benchmark model for litchi counting.

### Evaluation metrics

In the study of flower quantity estimation, 2 evaluation metrics are used: mean absolute error (MAE) and root mean squared error (RMSE). MAE quantifies the average discrepancy between the actual number and the predicted number of flowers in an image. RMSE, which is the square of the standard deviation, serves as a standard for assessing the efficacy of machine learning models. The formulas for these metrics are as follows:

$$\mathrm{MAE}=\frac{1}{N}\sum_{j}^{N}\left|z_{i}-{\hat{z}}_{i}\right|$$ (13)

$$\text{RMSE}=\sqrt{\frac{1}{N}\sum_{1}^{N}{\left(z_{i}-{\hat{z}}_{i}\right)}^{2}}$$ (14)

where *N* is the number of samples in the test set, *z_i_* is the number of labeled flowers in the *i*th image, ${\hat{z}}_{i}$ is the number of flowers predicted by the model for the *i*th image, MAE represents the accuracy of the prediction, and RMSE represents the robustness of the model.

In instance segmentation, average precision (AP) is a key metric used to assess performance. It measures the effectiveness of a model by considering various Intersection over Union (IoU) thresholds. These thresholds range from 0.5 to 0.95 in increments of 0.05, resulting in a total of 11 different IoU values. The AP is calculated for each threshold, representing the model’s performance at that threshold. AP is obtained by averaging the 11 AP values. Moreover, AP50 and AP75 are computed based on the IoU values comparing the ground truth masks with the predicted masks. If the IoU exceeds 50% or 75%, it indicates successful detection of the predicted mask. These metrics are valuable in assessing the accuracy and stability of algorithms across different IoU thresholds.

The coefficient of determination (*R*^2^) is an evaluation metric for measuring how well a statistical model predicts an outcome. In the study of flower quantification, it is applied to evaluate the relationship of flower number results between observation, annotation, and prediction. The higher the coefficient of determination *R*^2^, the higher the correlation. It is between 0 and 1. The formulas for these metrics are as follows, where *n* is the number of samples, ${\hat{Y}}_{i}$ is the *i*th prediction or annotation of flower number, *Y_i_* is the *i*th flower number observed, and ${\bar{Y}}_{i}$ is the average of all the flower number observed:

$$R^{2}=1-\frac{\sum_{i=1}^{n}{\left({\hat{Y}}_{i}-Y_{i}\right)}^{2}}{\sum_{i=1}^{n}{\left({\bar{Y}}_{i}-Y_{i}\right)}^{2}}$$ (15)

## Results

### Model performance and comparison

#### Ablation experiments of FlowerNet

To further investigate the effectiveness of FlowerNet, ablation experiments were conducted on the following 5 aspects:

1. Ablation study of FlowerNet

To minimize the influence of different image capture distances and backgrounds on flower quantity prediction, we implemented a multiscale feature fusion module and a mask branch module. Our experiments were carried out using a benchmark dataset comprising images of litchi captured at various distances and in natural environmental settings. The results are presented in Table 4. Our analysis demonstrates the pivotal importance of incorporating multiscale features into the FlowerNet, which enables the integration of feature maps at various scales to extract features from lychee flower images of different sizes. As a result, the accuracy of litchi flower quantity is significantly enhanced. Removing the multiscale features from the main network would diminish the network’s ability to comprehend the images and decrease its capacity to mitigate environmental interference, thereby reducing counting precision. Furthermore, the addition of a mask branch structure is advantageous for the model’s understanding of the distribution of litchi male flowers in the images and for achieving semantic segmentation of foreground and background, consequently enhancing the model precision. Failing to include the mask branch would lead to the model misinterpreting background features as flowers in the absence of semantic information, resulting in erroneous inclusion of background features.

**Table 4.** Ablation study experiment for multiscale feature fusion module and the mask branch structure. (The multiscale feature column indicates whether the experiment is conducted using only the feature maps outputted by the C2 layer of the backbone network or the panoramic feature pyramid model. Mask indicates whether mask branches are added, MAE and RMSE are evaluation indicators, and the best results are shown in bold.)

Multiscale feature	Mask	MAE	RMSE
		97.65	119.31
	√	90.47	104.25
√		70.72	90.41
√	√	**47.71**	**61.78**

2. Effectiveness analysis of different modules in head network

To examine the influence of various modules in head network on performance, a series of ablation experiments were carried out on the benchmark dataset in this section using the following networks:

(a) Baseline: The baseline network consists of VGG16, a panoramic feature pyramid, and a density mask generation network DMN. Simple “convolution-pooling” down-sampling operations are performed in both branches of the density mask generation network.

(b) Baseline+2MP: 2MP refers to the use of the MP module for down-sampling in both branches of the DMN part. In the experiment, the MP module is used for pooling operations on nonaligned feature maps in both branches.

(c) Baseline+MP: MP indicates that the MP module is used for down-sampling in the mask-generation branch of the DMN part, and simple “convolution-pooling” down-sampling is performed in the density generation branch.

(d) FlowerNet: In the DMN part, “convolution-pooling” down-sampling is performed on the mask generation branch, and the MP module is used for down-sampling feature extraction operations in the density generation branch.

According to the findings presented in Table 5, it is evident that the latter 3 networks show significant improvements in counting accuracy and exhibit satisfactory generalization performance compared to the baseline network. Specifically, for counting male litchi flowers, the MP module in the density mask generation network effectively represents the input feature map and performs favorably in extracting relevant information during the down-sampling phase. Furthermore, the mask generation network’s convolution-pooling down-sampling operation exhibits superior capabilities in extracting semantic information from the input hybrid feature map, thereby successfully accomplishing the mask segmentation task. Likewise, the results also demonstrate that both types of down-sampling operations possess a strong advantage in terms of interdependence.

**Table 5.** Effectiveness analysis results for different modules (the best results are shown in bold)

Models	MAE	RMSE
Baseline	63.04	76.90
Baseline+2MP	74.11	92.98
Baseline+MP	62.42	90.58
FlowerNet	**47.71**	**61.78**

3 .Panoramic feature pyramid output channel verification experiment

This section presents a verification experiment conducted on the output feature maps of the panoramic feature pyramid. In contrast to the original study’s proposal of setting the output channels in panoramic segmentation equal to the number of categories, this thesis utilizes this module for feature fusion at different scales, aiming to facilitate information sharing between features at different scales. Therefore, verification experiments with different numbers of output fusion feature map channels (128 and 256) to determine the optimal performance were conducted. In this experiment, FlowerNet is utilized as the baseline, and various channel configurations are examined. The results are presented in Table 6. It is evident from the results that setting the number of output channels to 256 enables the combination of low-frequency and high-frequency features in images within limited parameters, thereby enhancing the counting performance of the model.

**Table 6.** Experimental results of output channel verification for the panoramic feature pyramid (the best results are shown in bold)

Channels	MAE	RMSE
128	53.06	69.5
256	**47.71**	**61.78**

4. Ground truth mask dilatation verification experiment

To assess the impact of different mask areas on the flowers when expanded with structuring elements of different sizes within the model, the mask structure branch is implemented to effectively separate the foreground and background through fine-tuning. This, in turn, improves the model’s counting accuracy. In this experiment, FlowerNet was adopted as the benchmark, and a series of verification experiments were conducted to evaluate the model’s performance with ground truth mask expansion using structuring elements of different sizes. The experimental setup is detailed in Table 7. The results indicate that employing a suitably sized structuring element during mask expansion significantly aids the model in correctly distinguishing the foreground and background during training, consequently improving the precision in the segregation task. Notably, a 3×3 structuring element yields the best counting performance. Collectively, this experiment demonstrates the efficacy of fine-tuning the ground truth label with a 3×3 structuring element for the dataset used in this paper, enabling improved differentiation between the flower foreground and background.

**Table 7.** Verification experiment results for dilatation on ground truth masks. (Size represents the size of the structuring element, MAE and RMSE are evaluation indicators, None means non-usage of dilation, and the best results are shown in bold.)

Size	MAE	RMSE
None	64.83	86.75
**3×3**	**47.71**	**61.78**
5×5	74.76	112.65

5. Combined loss function ablation experiment

The proposed combined loss function is used to combine the mask loss and density map regression loss to optimize the model. It is crucial for the model to attain a balance between mask segmentation and density map regression to effectively complete the flower count task. This section presents an ablation experiment that investigates the impact of different values of α in the composite loss function on model performance. The results reveal that the model performs best when *α* is set to 8, leading to significant enhancements in counting accuracy and robustness, as shown in Table 8.

**Table 8.** Results of the combined loss function ablation experiment (the best results are shown in bold)

*α*	MAE	RMSE
2	88.23	108.07
4	74.27	90.84
**8**	**47.71**	**61.78**
10	70.95	92.16

#### Model evaluation

In this section, comparative experiments were conducted to evaluate the performance of instance segmentation and flower counting algorithms.

**Fig. 9.**
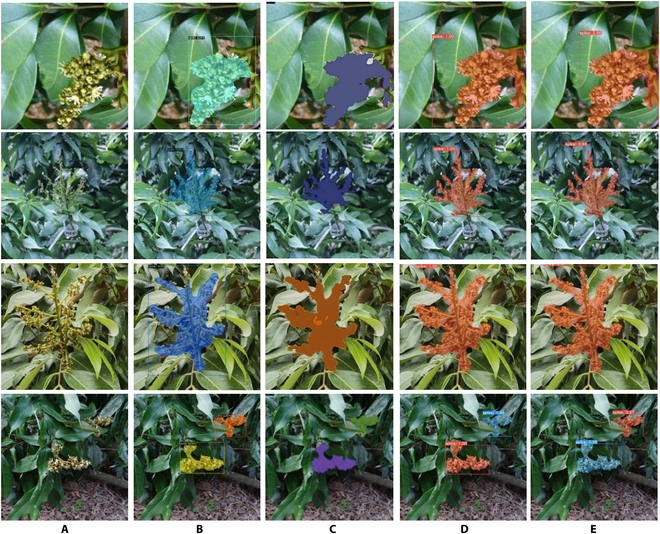
Various examples of instance segmentation results. (A) Original images, (B) mask R-CNN, (C) SOLOv2, (D) YOLACT, and (E) YOLACT++.

Firstly, various existing instance segmentation models were compared to identify a fast and suitable algorithm specifically for extracting flower panicles. Mask R-CNN [35], SOLOv2 [36], YOLACT [37], and YOLACT++ were selected. Among them, instance segmentation algorithms mainly fall into 2 categories. Mask R-CNN, as an advanced object detection-based 2-stage framework, maintains a precision advantage in many instance segmentation tasks. On the other hand, the SOLOv2 and YOLACT series algorithms are excellent single-stage instance segmentation algorithms based on global masking. SOLOv2 has the advantage of end-to-end processing, while YOLACT and YOLACT++ decompose the instance segmentation into parallel tasks, accelerating the instance segmentation speed of SOLOv2 and YOLACT series algorithms. Therefore, these 4 algorithms were chosen to validate the coarse detection process of litchi panicles. The results of these experiments are presented in Fig. 9 and Table 9. The average accuracy of the YOLACT and YOLACT++ for panicle extraction is similar to that of Mask R-CNN and the detection speed of single image of both models are better than others. The experimental results demonstrate that YOLACT++ effectively segments flower panicles and achieves overall satisfactory segmentation outcomes. This model proves to be suitable for accurately segmenting individual flower panicles in close-range scenes, meeting the requirements for flower panicle extraction in this study.

**Table 9.** Various model performance results (the best results are shown in bold)

Models	Time (s)	AP	AP50	AP75
Mask R-CNN	0.07	47.5	**89.5**	47.1
SOLOv2	0.06	22.9	61.4	16.5
YOLACT	**0.01**	47.9	82.3	**58.4**
YOLACT++	0.02	**48.3**	84.8	54.1

**Fig. 10.**
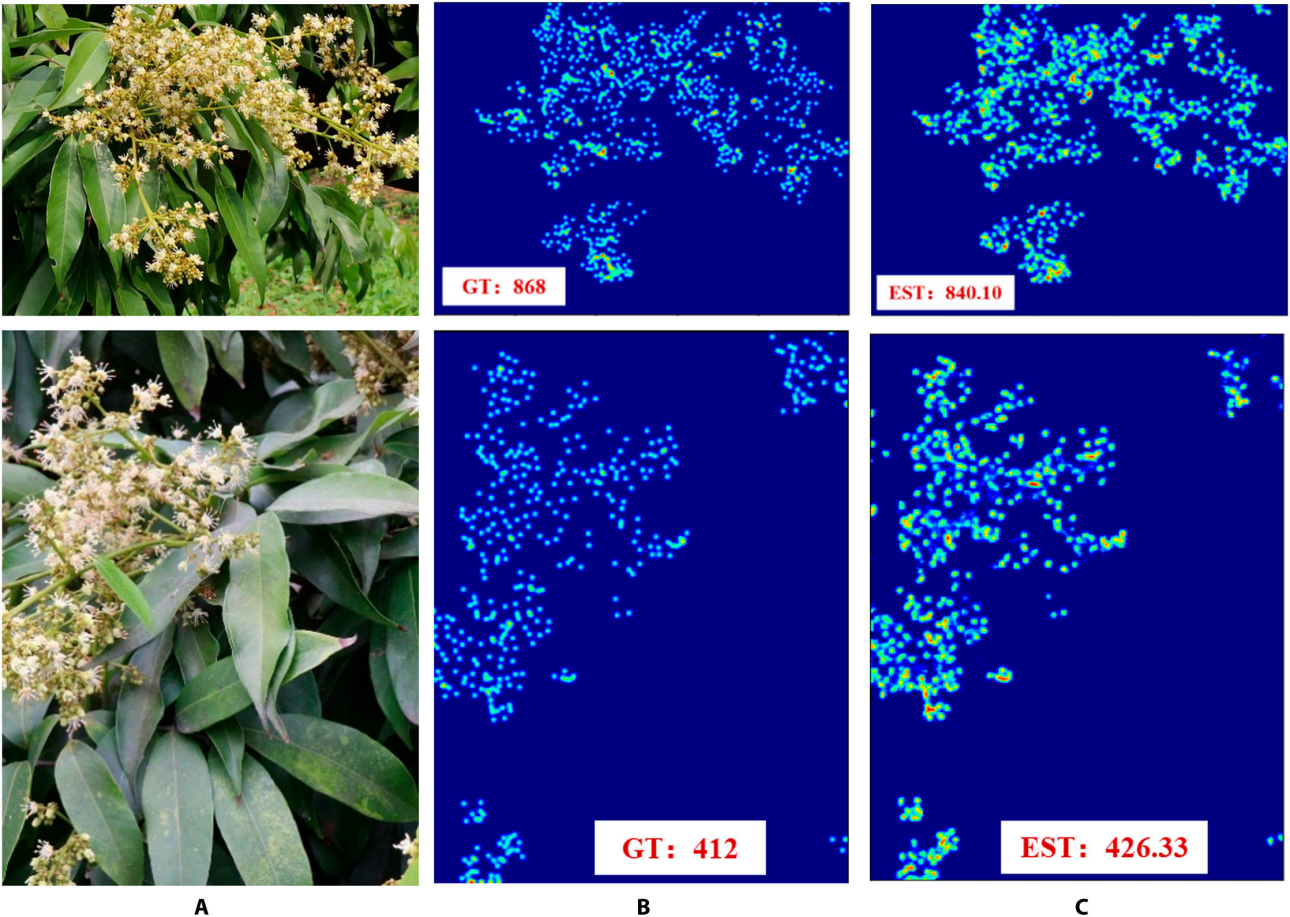
Examples of flower number prediction results from FlowerNet. The columns are (A) original images, (B) ground truths, and (C) predictions.

An example flower counting prediction result of FlowerNet is shown in Fig. 10. FlowerNet using the density map regression counting method can complete the counting of litchi flowers well. At the same time, in the density map predicted by the algorithm, there is basically no counting in the non-flower area of the input image. The predicted density map shows the approximate spatial distribution of litchi flowers well.

#### Comparative performance of counting algorithms

The performance of existing flower counting models based on density map regression was analyzed using the multicolumn CNN (MCNN) proposed by Zhang et al. [38], the 5-column neural network (FCNN) proposed by Lin et al. [29], and the CSRNet proposed by Li et al. [39]. The first 2 types of networks analyze the features of input images with convolution kernels of different sizes and complete the task of density map regression. CSRNet is composed of 2 major components: a feature extraction network VGG16 and a dilated CNN for the back end. Table 10 compares various indicators of FlowerNet and these 3 types of flower counting algorithms. MAE and RMSE represent the performance of the model. FLOPS (floating point operations per second) is a measure of the performance of a computer processor. It represents the number of floating-point operations the processor can perform per second. Params (parameters) in the CNN refers to the total number of parameters of all layers in the model, including the convolutional layer, pooling layer, and fully connected layer, which can be used to measure the complexity and capacity of the model.

**Table 10.** Performance comparison of algorithms (the best results are shown in bold)

Model	MAE	RMSE	FLOPS	Params
MCNN	71.76	88.84	**8.6321G**	**0.1280M**
FCNN	61.37	77.24	44.1389G	1.0256M
CSRNet	51.99	66.96	169.1619G	16.2635M
FlowerNet	**47.71**	**61.78**	234.3044G	19.0102M

As shown in Table 10, the network model FlowerNet designed in this chapter achieves the best MAE and RMSE values in solving flower counting problems of different forms during the litchi flowering period. On the male litchi flower dataset, the MAE of FlowerNet is reduced by 24.05 and the RMSE value is reduced by 27.06 compared with those of MCNN; the counting performance index MAE is reduced by 13.66 and the RMSE value is reduced by 16 compared to those of the FCNN. CSRNet delivers an MAE of 51.99 and an RMSE of 66.96. By comparison, it is observed that FlowerNet shows significantly improved performance on datasets with large changes in the morphology of male litchi flowers, proving the effectiveness of FlowerNet.

**Fig. 11.**
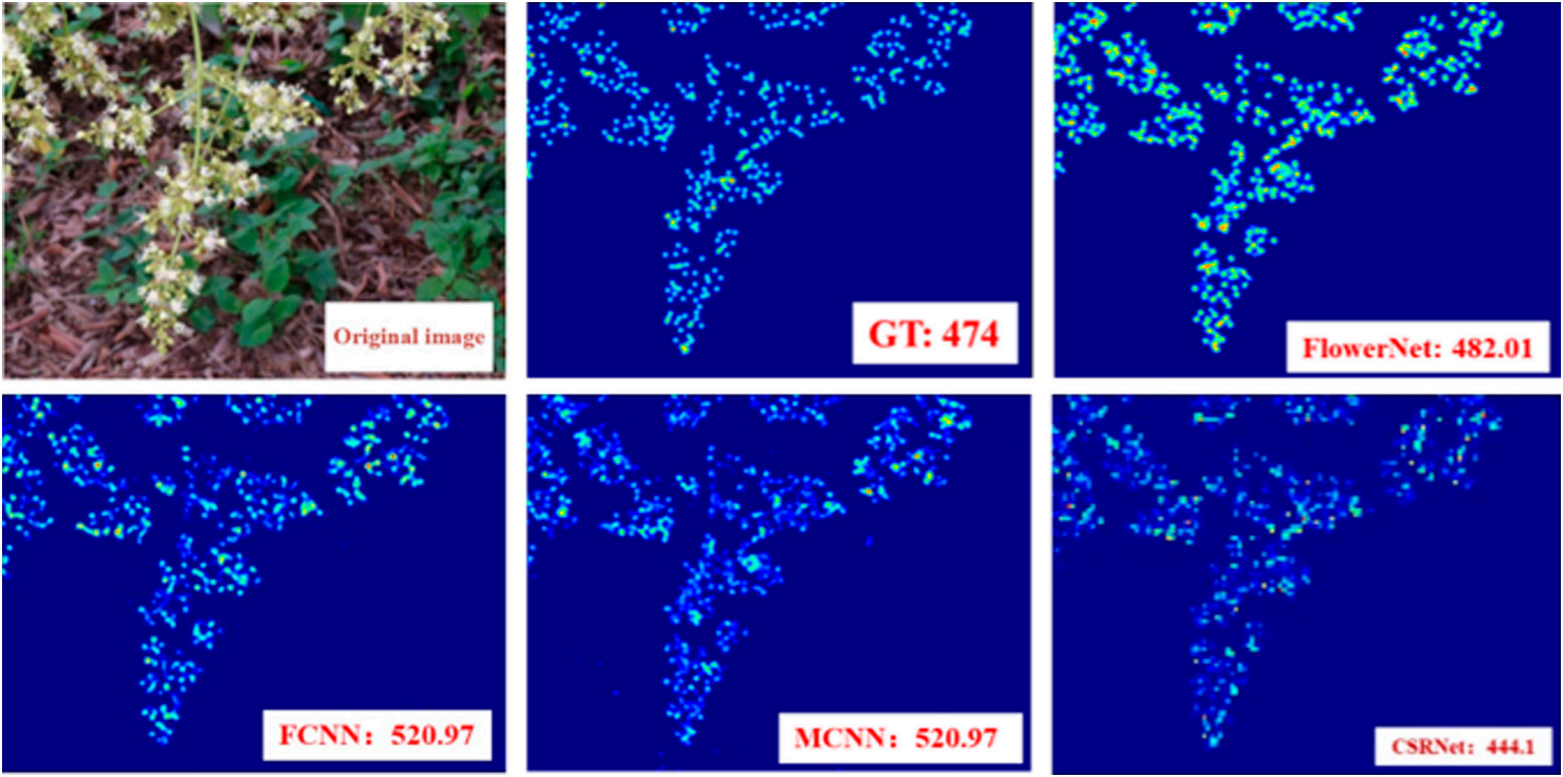
Example comparison of predicted density maps for different models.

Figure 11 compares the predicted density maps of FlowerNet and other networks on this male litchi flower counting dataset. Against many backgrounds, both the MCNN and FCNN algorithms suffer from counting errors in the detection area, while the FlowerNet proposed in this chapter shows the best performance against complex backgrounds, further demonstrating the superiority of this algorithm.

### Flower quantification

#### Regression analysis of flower quantity

This section used Origin 2018 to perform regression analysis on actual flower counts, manually counted flower counts from images, and predicted flower counts from FlowerNet. First, the Pearson correlation coefficient algorithm was employed to calculate the correlation coefficients separately and conduct a significance test. The correlation coefficient measurement results are presented in Table 11. From the correlation test between the image flower count and the actual flower count, it is evident that the actual count of single-panicle flowers is highly positively correlated with both the manually annotated image count (*P* < 0.01, *r* = 0.941) and the predicted flower count from the FlowerNet model (*P* < 0.01, *r* = 0.907). By means of the correlation analysis test, it is determined that there exists a robust correlation between the actual flower count and the image annotation flower count, as well as between the actual flower count and the predicted flower count. This relationship can be verified through regression analysis.

**Table 11.** The results of the correlation test. (FL represents the number of flowers counted in the image, FF represents the number of flowers predicted by FlowerNet, and FH represents the real number of flowers per panicle counted by hand.)

Parameter	FH
FL	0.941
FF	0.907

**Fig. 12.**
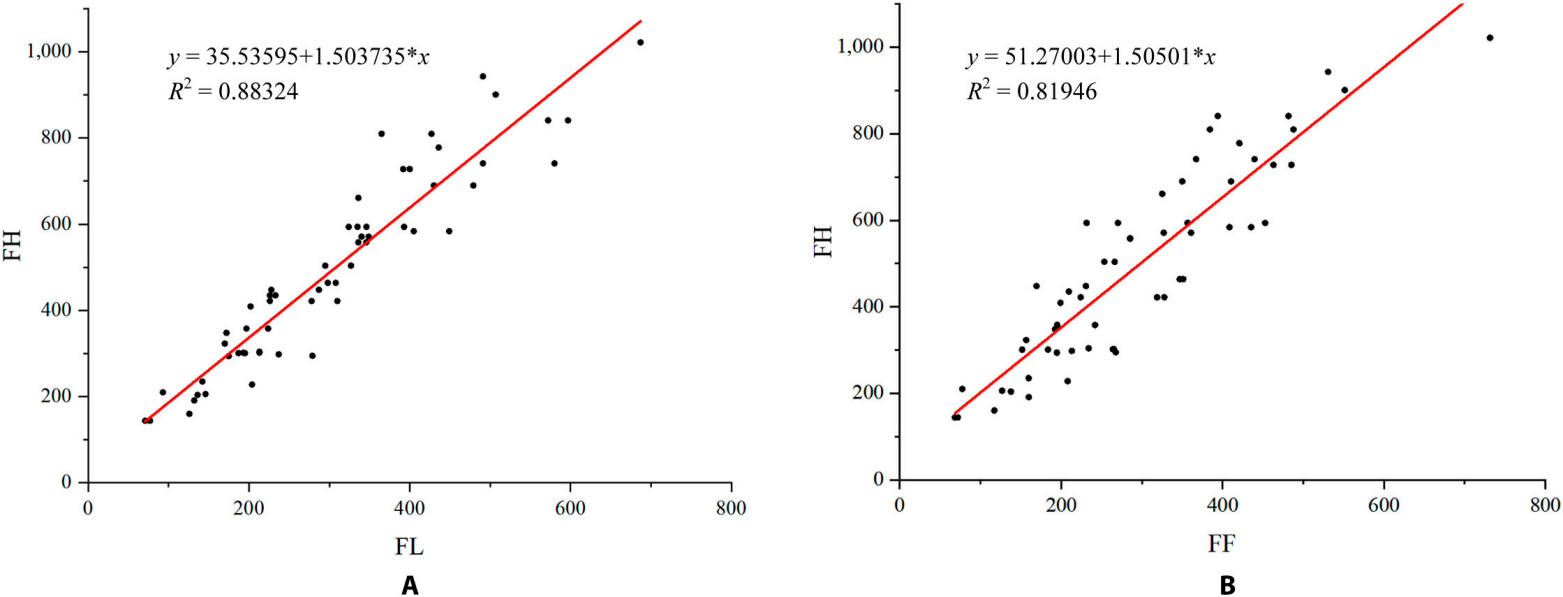
Linear fitting regression results. (A) The regression result of the flower number on the image and the actual flower number. (B) The regression result of the flower number predicted by FlowerNet and the actual flower number. FL represents the number of flowers counted in the image, FF represents the number of flowers predicted by FlowerNet, and FH represents the real number of flowers per panicle counted by hand.

According to the manual annotation results for counting flower images, a linear regression equation was obtained by fitting the manually counted actual flower count data. This regression equation correlates image flower counts with actual counts of flowers taken from normal shot angles, as shown in Fig. 12A. Images of different flower panicles, captured from a side view, show a reasonable fit with the actual single-panicle flower count, with a coefficient of determination of 0.88. This indicates that the regression equation derived from the image flower counts can be used to estimate the corresponding actual flower count for a single panicle. The image flower count prediction accuracy can be improved through modeling, enabling the description of the actual flower count of individual Freesia flower panicles at the initial flowering stage. The regression equation is expressed as *y* = 35.53595 + 1.503735*x*.

To improve the accuracy of the model’s predictions, the ability of the FlowerNet model to estimate the count of single-panicle flowers is investigated. The model was trained using a fixed background board as the detection background. It then predicts the actual flower count in the sample and establishes a linear regression equation based on the true flower count. As shown in Fig. 12B, the FlowerNet model estimate of the actual flower count has a coefficient of determination of 0.81. This suggests that the model is able to effectively predict the flower count of a single panicle, making it suitable for automation in flower count estimation. The regression fitting equation is *y* = 51.27003 + 1.50501 ∗ *x*.

#### Verification experiment for the linear model

In the experiment for verifying the linear model, 10 samples of single-panicle images, along with their corresponding results from manual counting, were obtained. These specific sample images were input into FlowerNet to predict the number of flowers in each image. The predicted results were then compared with the manual counting values by estimating and fitting a regression equation. This comparison enables us to evaluate the accuracy of the flower count per panicle using the following formula:

$$P=\left(1-\frac{\left|p_{\text{Ground truth}}-p_{\text{Predict}}\right|}{p_{\text{Ground truth}}}\right)\times 100\%$$ (16)

where *P* is the accuracy rate, *p_Ground truth_* is the manually counted number of flowers per panicle, and *p_Predict_* is the flower quantity prediction result of the model.

Table 12 presents the results of the analysis on a set of 10 randomly selected samples. The combination of the FlowerNet model and linear regression fitting equation is used to predict the actual number of flowers of a single panicle. The overall accuracy of this prediction is determined to be 85.59%. Furthermore, MAE and RMSE are found to be 49.4 and 59.57, respectively. Importantly, the estimation device used in this experiment may introduce errors into the actual estimation results for the number of flowers per panicle. This is because the total flower quantity per panicle prediction is obtained indirectly through the linear regression model, and the prediction error is a combination of the error caused by the regression model and the error caused by the FlowerNet.

**Table 12.** Verification of the number of flowers. (FH represents the real number of flowers per panicle counted by hand, FM represents the number of flowers predicted by FlowerNet and the linear model, Difference is the difference between FH and FM, *P* is the accuracy rate, and Average is the average accuracy rate.)

Index	FH	FM	Difference	*P*
1	221	284	63	71.49%
2	348	350	2	99.43%
3	240	299	59	75.42%
4	204	259	55	73.04%
5	144	154	10	93.06%
6	301	328	27	91.03%
7	464	573	109	76.51%
8	571	544	27	95.27%
9	778	685	93	88.05%
10	728	782	54	92.58%
MAE	–	–	–	49.9
RMSE	–	–	–	59.57
Average	–	–	–	85.59%

## Discussion

Information regarding flowering can be indicative of the growth of litchi fruit trees. Currently, manual counting is the predominant method used to gather phenotypic information on litchi flower flowering. However, this approach is time-consuming and requires significant labor. With advancements in computer vision and deep learning, researchers have started utilizing these technologies to enhance the efficiency of flower counting. Traditional machine learning algorithms have employed different color thresholds in images to calculate the pixel area of flowers, subsequently mapping the flower count based on the extracted pixels. Nevertheless, this method necessitates stringent control of the lighting conditions at the data collection site [10]. Moreover, some researchers have employed deep learning techniques, such as object detection and instance segmentation [17,18]. This involves extracting the targets in the images, resulting in an accurate flower count. However, detecting complex targets such as various litchi flowers poses challenges, including difficulties in object annotation and model detection compared to traditional fruit detection methods. During annotation, attention must be devoted to precisely determining the size and location of the object to obtain accurate measurements of flowering in the images. Additionally, researchers have implemented density map regression to count litchi flowers. This method directly obtains the flower count through the model without requiring predictions of target size or location [29]. Nevertheless, density map regression, being a pixel-level task, is highly sensitive to input image information and may introduce noise in nontarget areas, thereby reducing the count accuracy.

This paper proposes a framework for accurately counting the number of litchi flowers using the instance segmentation and multitask learning approaches. The estimation of individual flower panicle quantities in the images can be achieved by combining 2 different algorithms. In this context, a novel algorithm for counting flower quantities is proposed. This model can directly learn the relationship between litchi flower images and the number of flowers present. The output density map effectively retains the spatial position information of the target flowers, enabling it to provide valuable insights such as the approximate spatial distribution of the flowers. This information can be utilized to improve the model results and identify abnormal situations. Additionally, the model can learn 2 tasks simultaneously: background distinction and flower counting. By sharing key information from both tasks, the model effectively removes the interference caused by background elements from the count results.

**Fig. 13.**
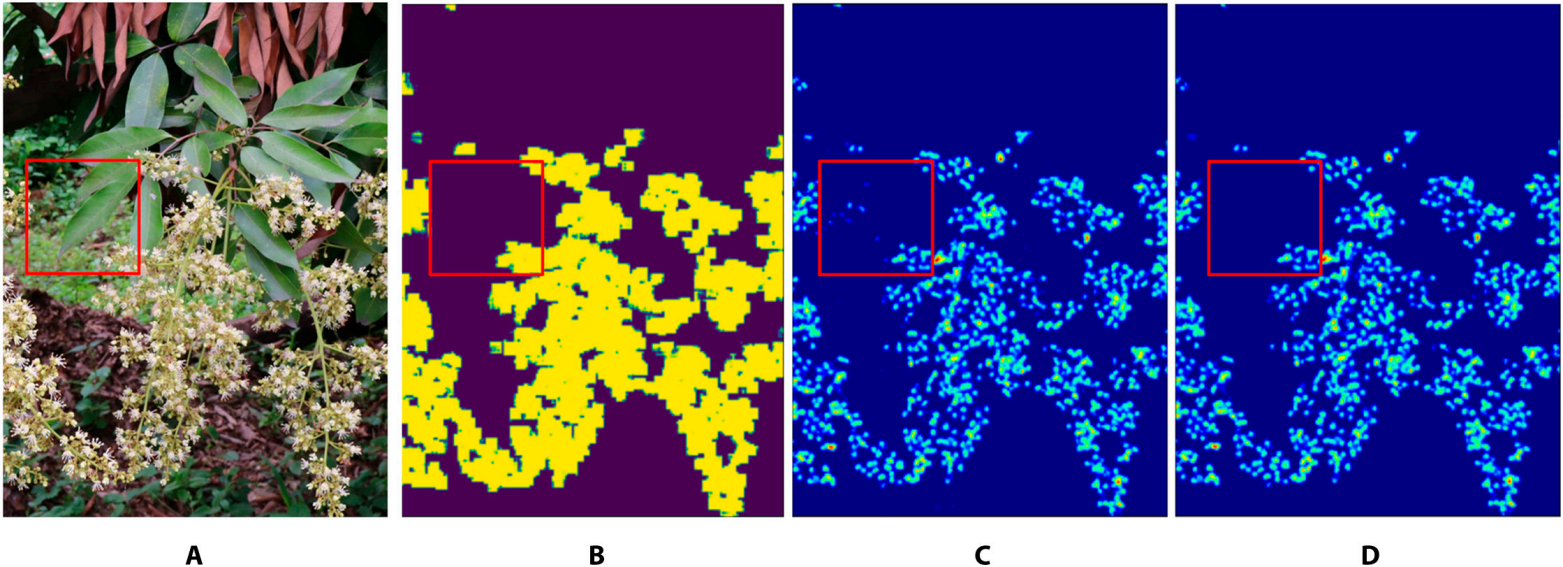
Example analysis of the prediction results of FlowerNet. (A) The input image, (B) the mask generated by the mask generation module, (C) the density map generated by the density generation module, and (D) the final density map after prediction.

The FlowerNet algorithm shows good performance in image flower counting, as shown in Fig. 11. In particular, the proposed method can significantly distinguish between the front and back backgrounds in different images, as shown in Fig. 13. The generated mask helps the predicted density map remove regions with incorrect counts, which demonstrates that adding the mask generation module obviously improves the counting result. This method learns the regression of the flower density map and the distinction of the background mask and the corresponding density map regression through a large amount of dataset training. At the same time, the reasonableness of the parameter settings of the network module in this paper is verified through different ablation experiments. FlowerNet, as detailed in this paper, was utilized to investigate a specific application scenario in which the precise number of flowers per litchi panicle was estimated through linear fitting analysis. The 2-dimensional image data of individual panicles provided valuable information on the actual flower count, thereby enabling reliable estimation of the flower quantity per panicle.

To examine the limitations of the FlowerNet algorithm, it is evident from the validation set’s performance that the counting model requires significantly greater FLOPS than the other 2 models. This disparity suggests a relatively high complexity in the algorithm model. Consequently, on the same hardware platform, FlowerNet requires a longer processing time for flower counting. Moreover, when counting the number of flowers on a single flower panicle using a 2-dimensional image alone, the flowers concealed beneath the leaves are not counted. The provided table demonstrates that FlowerNet still maintains a certain degree of accuracy in calculating the actual flower count. This finding suggests that relying solely on image information from a single perspective is insufficient to comprehensively reflect the actual flower count for a single panicle. To address the challenge of occlusion when automatically estimating the true number of flowers, future research should explore the utilization of images captured from multiple camera views and the integration of predictions from each view to achieve more precise flower count results.

Consequently, our study established the framework for predicting the phenotypic information parameters of flowering number in litchi trees during the flowering period in fruit orchards.

The implementation of a framework for counting individual flower panicles has been completed. The framework utilizes the real-time instance segmentation algorithm YOLACT++ to efficiently extract individual flower panicles. Once the mask results are obtained, they are fed into the proposed new counting algorithm, FlowerNet, to accurately determine the quantity of flower panicles.

Based on the VGG-16 backbone feature extraction network, a new method, FlowerNet, was highlighted that directly estimates the flower count from images of litchi flowers. Compared to other methods in the literature, this model showed better counting performance on the litchi flower density map dataset constructed, obtaining an MAE of 47.71 and an RMSE of 61.78.

 The task of foreground and background segmentation was introduced into the network model, generating 2 types of masks for flowers and backgrounds. This enables the network to differentiate the semantic information of the image in the density map counting task, reducing background interference with the results. In the generation of the predicted density map, the generated mask results are shared, reducing the counting error more effectively.

 In the construction of dataset labels, based on the characteristics of different forms of litchi flowers in the same flowering period, a full-flowering period male flower image dataset was constructed. Morphological operations were introduced, and dilation operations were used to fine-tune the size of the mask foreground to meet the requirements of foreground and background separation tasks for litchi flowers of different forms.

 Using the proposed FlowerNet model and based on the single-panicle flower count dataset, the count of single flower panicles was estimated. A linear regression model for single-panicle flower count estimation was established based on the flower count results predicted by the algorithm. The final coefficient of determination was 0.81, further improving the counting accuracy.

The method proposed in this paper can provide a useful reference for estimating the count of small and dense flowers in the future. While the proposed method was found to effectively estimate the flower count of litchi in dense scenes, these results were obtained under a limited sample capacity. In the future, it will be necessary to add more datasets under natural scenes and introduce research on flower count estimation from multiple perspectives to obtain the phenotypic information parameters of litchi flowering more accurately and automatically. Besides, the accuracy and stability of single-panicle segmentation can still be improved.

## Data Availability

Some data and code that were used in this study have been uploaded to the website https://github.com/Chiaquan/Multi-task-Litchi-Flower-Counting. In addition, all the litchi flower counting dataset in this study can be obtained from the corresponding author upon reasonable request.
